# Salicylate-Induced Hearing Loss and Gap Detection Deficits in Rats

**DOI:** 10.3389/fneur.2015.00031

**Published:** 2015-02-20

**Authors:** Kelly E. Radziwon, Daniel J. Stolzberg, Maxwell E. Urban, Rachael A. Bowler, Richard J. Salvi

**Affiliations:** ^1^Department of Communicative Disorders and Sciences, Center for Hearing and Deafness, University at Buffalo, The State University of New York, Buffalo, NY, USA; ^2^Department of Physiology and Pharmacology, Schulich School of Medicine and Dentistry, University of Western Ontario, London, ON, Canada

**Keywords:** gap detection, operant conditioning, threshold, tinnitus, prepulse inhibition, rat model, sodium salicylate, sensorineural hearing loss

## Abstract

To test the “tinnitus gap-filling” hypothesis in an animal psychoacoustic paradigm, rats were tested using a go/no-go operant gap detection task in which silent intervals of various durations were embedded within a continuous noise. Gap detection thresholds were measured before and after treatment with a dose of sodium salicylate (200 mg/kg) that reliably induces tinnitus in rats. Noise-burst detection thresholds were also measured to document the amount of hearing loss and aid in interpreting the gap detection results. As in the previous human psychophysical experiments, salicylate had little or no effect on gap thresholds measured in broadband noise presented at high-stimulus levels (30–60 dB SPL); gap detection thresholds were always 10 ms or less. Salicylate also did not affect gap thresholds presented in narrowband noise at 60 dB SPL. Therefore, rats treated with a dose of salicylate that reliably induces tinnitus have no difficulty detecting silent gaps as long as the noise in which they are embedded is clearly audible.

## Introduction

The ototoxic effects of high doses of salicylate, the active ingredient in aspirin, are well-established (1–4). A recent review by Sheppard et al. (5) details the peripheral and central effects of salicylate along with the known perceptual deficits seen in humans following large doses of aspirin (5). Peripherally, acute doses of salicylate affect outer hair cell (OHC) and auditory nerve function, causing a reduction in distortion product otoacoustic emissions (DPOAE) and cochlear compound action potentials (CAP), respectively (6, 7). Centrally, systemic administration of salicylate amplifies sound-evoked activity in the auditory cortex (AC) to high-intensity sounds despite reduced neural output from the cochlea (8, 9). Also, salicylate alters spontaneous firing rates (10) and tonotopic organization in the AC (7).

In addition to electrophysiological studies, some of the perceptual effects of salicylate have been cataloged. Depending upon the dose, humans and rats typically develop hearing loss, with temporary threshold shifts of ~5–20 dB SPL, following salicylate administration (1, 2, 11, 12). In addition to hearing loss, salicylate also disrupts auditory temporal processing for normal-hearing human listeners. With moderate doses of aspirin, McFadden et al. (13) found increased gap detection thresholds (impaired temporal resolution) at low sound-pressure levels and flatter than normal temporal integration functions (impaired temporal integration). These aspirin-induced changes in temporal resolution and temporal integration are similar to the temporal processing deficits seen in listeners with sensorineural hearing loss (14, 15). Thus, aspirin-induced hearing loss has been proposed as a model of temporary sensorineural hearing loss in humans (13).

In addition to inducing temporary hearing loss and temporal processing deficits, salicylate also reliably induces tinnitus, i.e., a phantom auditory perception, in humans (2). Given its tinnitus-inducing properties, salicylate was used to test the first animal model of tinnitus (16), and most of the current animal behavioral models of tinnitus have been assessed using salicylate [see Ref. (17, 18) for recent reviews of animal models of tinnitus]. Regarding behavioral models of tinnitus, one of the most widely used animal behavioral paradigm for tinnitus uses a modified gap detection paradigm. Based on the hypothesis that tinnitus fills in the silent gap, the gap prepulse inhibition of the acoustic startle reflex (Gap-PPI) paradigm has been used to assess both salicylate- and noise-induced tinnitus in a number of species (19–25).

In a typical Gap-PPI paradigm, a 50 ms silent interval embedded in 60 dB SPL continuous noise is presented ~100 ms before a high-intensity noise burst used to evoke the startle reflex motor response (26, 27). Normal subjects hear the silent gap, which functions as a warning signal (cue) that the startle stimulus is about to occur, and reduces the amplitude of the startle reflex. However, the “tinnitus gap-filling” hypothesis postulates that if a subject has tinnitus, the phantom sound “fills in silence” and interferes with processing of the silent gap, thereby preventing the silent interval from inhibiting the startle reflex. The greatest deficit in Gap-PPI presumably occurs when the spectral properties of the background noise match the tinnitus pitch (28–30). Consequently, narrowband noise (NBN) Gap-PPI has been used to estimate the tinnitus pitch (19, 29, 31).

Gap-PPI has been widely used by many researchers, including our group, to identify animals that presumably have noise- or drug-induced tinnitus (19–21, 23). Gap-PPI paradigms attempt to avoid the problem of drug- or noise-induced impairments in temporal resolution by employing 50 ms gaps presented in at least 60 dB SPL background noise, durations and intensities that are typically well above threshold. Therefore, researchers have assumed that impaired Gap-PPI is not due to impaired temporal resolution *per se*, but rather to tinnitus interfering with the processing of the silent gap (24).

However, since the effect of salicylate on auditory temporal resolution has not been directly investigated in rats, it is not clear whether temporal processing deficits, hearing loss, tinnitus, or some other perceptual deficit can explain changes in Gap-PPI performance following salicylate administration (1, 32). Therefore, the goal of the current study is to determine the effects of salicylate on conscious gap detection performance in rats.

To evaluate basic temporal resolution in rats, we treated rats with a dose of salicylate (200 mg/kg IP) known to induce tinnitus (4) and obtained gap detection thresholds for broadband and NBNs. If the 50 ms gaps in 60 dB background noise cannot be detected after salicylate treatment, then this would support the hypothesis that tinnitus fills in silent intervals in their conscious perceptions. However, if gap detection thresholds remain largely unchanged following salicylate administration, then it is unlikely that tinnitus is filling in the silent interval and preventing the 50 ms gap from being consciously perceived. Although the conscious perception of silent gaps in psychoacoustic tasks is likely different than the mechanisms involved in the subcortical Gap-PPI paradigm, this study will provide important information for evaluating what is modeled in animal models of tinnitus, i.e., hearing loss, temporal processing deficits, or hyperacusis (32).

## Materials and Methods

### Subjects

Six female Sprague-Dawley rats were used in the gap detection experiments, and three of these six rats were used to obtain noise thresholds (Note: two rats developed tumors and had to be sacrificed before their gap detection thresholds in NBN could be obtained, and one rat had a broken tooth preventing her from being used during the NBN detection experiment). The rats began training at ~4 months of age and they were tested until they were ~14–15 months of age. The rats were obtained from Charles River Laboratories and all procedures were approved by the University at Buffalo, SUNY’s Institutional Animal Care and Use Committee. All rats were housed separately and were kept on a 12 h day/night cycle (lights on at 6 a.m.; lights off at 6 p.m.). Rats were food restricted and kept at ~85% of their free-feeding weight during the course of the experiment. Test sessions lasted ~1 h, and the rats were run once per day, 6–7 days/week.

### Gap detection procedures

#### Apparatus

Rats were tested in an acoustically transparent acrylic cage (28 cm × 30 cm × 38 cm) located inside a sound attenuating chamber (76 cm × 71 cm × 71 cm) lined with 5 cm thick sound attenuating foam (Illbruck, Inc., Minneapolis, MN, USA). The behavior of the animals during test sessions was monitored by a digital camera (Fire-i Digital Camera, Unibrain, San Ramon, CA, USA). The test cage was equipped with a speaker (FT28D Dome Tweeter, Fostex, Tokyo, Japan), feeder (Med Associates Model ENV-203M, St. Albans, VT, USA), and nose-poke hole equipped with infrared sensors (Vulintus, Dallas, TX, USA).

The experiment was run using custom behavioral software running on a personal computer (Microsoft Windows XP) similar to that described previously (4). The custom software controlled Tucker-Davis Technologies (TDT, Gainesville, FL, USA) system-3 equipment and operant hardware. Sound stimuli were generated with TDT hardware and software (TDT RX6 processor, D/A converter, ~100 kHz sampling rate); digital inputs to and outputs from the testing cages were controlled by the TDT RX6 processor interfaced to a pellet feeder (Med Associates Model SG-501, St. Albans, VT, USA) and infrared sensors (Vulintus, Dallas, TX, USA). TDT RPvds software and custom MATLAB software (MathWorks, Nattick, MA, USA) were used to control all aspects of the experiment. Sound-pressure levels were calibrated using a sound level meter (Larson-Davis System 824) equipped with a microphone (1/4″ free field microphone, model 2520, Larson-Davis, Depew, NY, USA) placed at the position where the animal’s head would be when it poked its nose into the nose-poke hole.

#### Procedure

The stimuli used in the gap detection experiments were a continuous broadband noise (BBN) containing frequencies up to 42 kHz, and 3 continuous NBNs: 10–20 kHz (1 octave bandwidth, centered at 14.14 kHz), 16–20 kHz (4 kHz bandwidth), and 15.3–16.7 kHz (1/8th octave bandwidth centered at 16 kHz). The BBN was presented at 20, 30, 40, and 60 dB SPL, and the NBNs were presented at 60 dB SPL. The rats were trained using a go/no-go operant conditioning procedure to detect a silent gap embedded within the continuous noise.

The rat began a trial by nose poking through the *nose-poke hole*, which initiated a variable waiting interval ranging from 1 to 4 s. During this time, the rat had to maintain its position in the nose-poke hole; failure to do so resulted in an aborted trial. After the waiting interval, a single silent gap was presented in the noise. In the *go* condition, the target stimulus was the silent gap. In this trial type, a *hit* was recorded if the rat correctly responded to the gap within 2 s by removing its nose from the nose-poke hole and receiving a food pellet (45 mg dustless rodent grain pellets, Bio-Serv, Frenchtown, NJ, USA). A *miss* was recorded if the rat failed to remove its nose from the nose-poke.

Approximately 30% of all trials were *catch* trials. These constituted the *no-go* part of the procedure and the silent gap was absent during these trials. False alarm (FA) rates and response biases were calculated from these catch trials. If the rat removed its nose during a *catch* trial, a *false alarm* was recorded and the rat received a 4 s timeout, during which the house light was turned off and the rat could not initiate another trial. However, if the subject continued to nose-poke, a *correct rejection* was recorded. No reinforcement was given for a *correct rejection*, but the next trial began immediately. Chance performance was represented by the animal’s *false alarm* rate. Sessions were excluded from analysis if the percentage of FAs was >20%. Less than 1% of all sessions were excluded for this reason.

The target stimuli were presented according to the psychophysical method of constant stimuli (MOCS). Within each 10-trial block, 7 predetermined targets were presented randomly along with 3 *catch* trials. During testing, the target gaps were chosen so that the smallest one or two gaps were rarely detected by the rats, whereas the largest gaps were well above threshold and nearly always detected. The gap durations used for testing were adjusted depending on each rat’s performance.

#### Sodium salicylate administration

After baseline gap detection thresholds were collected, the rats were tested once per week with either a single injection of sodium salicylate (200 mg/kg IP) dissolved in saline (50 mg/ml), or an equivalent volume of saline (control). The injections were administered 2 h before testing. The 200 mg/kg dose of sodium salicylate has previously been shown to reliably induce tinnitus-like behavior in rats (4, 33).

#### Data analysis

Signal detection analysis was employed to factor out the animals’ motivational biases (34). Mean *hit* and *false alarm* rates were used to calculate all gap thresholds using signal detection theory and a threshold criterion of *d*′ = 1.5. This *d*′ value is comparable to that used by others (35–37) and ensures that the animal is responding primarily to the target stimuli and not randomly (38).

### Noise detection procedures

#### Apparatus

The apparatus for this part of the study was the same as that used in the gap detection experiment.

#### Procedure

The spectra of the noise-burst stimuli used in the noise detection experiments were the same as in the gap detection study, namely, BBN and the three NBNs (10–20, 16–20, and 15.3–16.7 kHz). Each noise stimulus was 300 ms in duration with cosine rise/fall times of 5 ms. Rats were trained using a go/no-go operant conditioning procedure to detect a noise burst in an otherwise quiet chamber.

The procedure for obtaining the noise-burst thresholds was nearly identical to the procedures used in the gap detection experiment, but the target stimuli in the *go* condition for this experiment were noise bursts embedded in a quiet background. Approximately 30% of all trials were *catch* trials, and no stimulus was presented on *no-go* trials. Noise-burst threshold testing began when the animals finished the gap detection experiment. As in the gap detection experiment, noise bursts were presented using the MOCS. The target intensities were chosen so that only the lowest one or two intensities were not detected whereas the highest sound levels were well above threshold.

#### Sodium salicylate administration

After baseline noise-burst thresholds were collected, the rats were tested once per week with either a single injection of sodium salicylate (200 mg/kg IP) dissolved in saline (50 mg/ml) or an equivalent volume of saline (control). As in the gap detection experiments, the injections were administered 2 h before testing.

#### Data analysis

The data analysis for this study was the same as that used in the gap detection experiment.

## Results

### Gap detection

#### Gap detection in broadband noise

Table 1 shows the individual and mean gap detection thresholds in BBN at 30, 40, and 60 dB SPL for baseline, saline, and salicylate conditions along with gap detection thresholds for 20 dB SPL for baseline and saline conditions. Data could not be collected for the 20 dB SPL-salicylate condition because the rats were unable to perform the task. This was most likely due to the salicylate-induced hearing loss that resulted in the 20 dB BBN being below or near the threshold of audibility as determined in the subsequent threshold detection experiment (Table 1).

**Table 1:** **Individual and mean broadband noise gap thresholds (ms) vs. intensity (dB SPL) for baseline, saline, and salicylate conditions**.

Subject	Condition	BBN (60 dB SPL)	BBN (40 dB SPL)	BBN (30 dB SPL)	BBN (20 dB SPL)
Green 1	Baseline	2.1 ms	2.1 ms	2.7 ms	3.7 ms
	Saline	1.8 ms	2.3 ms	2.2 ms	3.8 ms
	Salicylate	2.0 ms	2.9 ms	11.3 ms	NC
Green 2	Baseline	1.3 ms	1.6 ms	2.4 ms	2.9 ms
	Saline	1.7 ms	1.8 ms	2.7 ms	5.7 ms
	Salicylate	1.5 ms	2.6 ms	3.9 ms	NC
Green 3	Baseline	1.8 ms	1.7 ms	2.7 ms	3.9 ms
	Saline	1.9 ms	2.0 ms	1.7 ms	6.9 ms
	Salicylate	NA	3.8 ms	NA	NC
Purple 1	Baseline	1.9 ms	2.1 ms	2.7 ms	3.3 ms
	Saline	2.3 ms	2.0 ms	2.4 ms	3.7 ms
	Salicylate	2.2 ms	2.5 ms	5.2 ms	NC
Purple 2	Baseline	1.5 ms	2.4 ms	3.0 ms	7.9 ms
	Saline	1.7 ms	2.6 ms	2.8 ms	9.7 ms
	Salicylate	1.6 ms	4.5 ms	17.7 ms	NC
Purple 3	Baseline	1.8 ms	1.8 ms	2.9 ms	4.4 ms
	Saline	2.2 ms	1.5 ms	2.5 ms	6.5 ms
	Salicylate	1.4 ms	3.0 ms	12.0 ms	NC
Mean	Baseline	1.72 ms	1.94 ms	2.71 ms	4.32 ms
	Saline	1.93 ms	2.03 ms	2.38 ms	6.05 ms
	Salicylate	1.74 ms	3.22 ms	10.02 ms	NC

*NA, data not available; NC, data not collected*.

#### Effect of intensity on BBN gap thresholds

**Figure 1:**
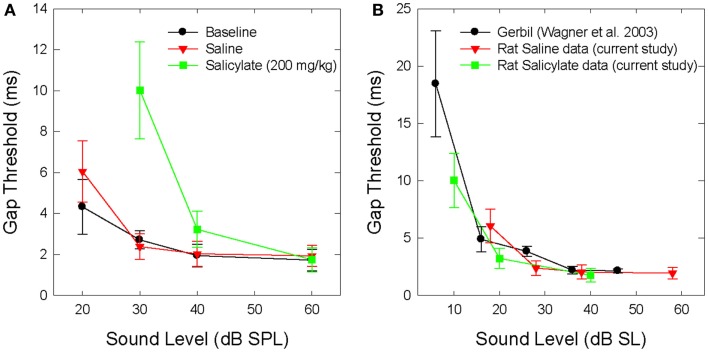
**(A)** Mean (SEM) gap thresholds for BBN presented at intensities from 20 to 60 dB SPL for baseline, saline, and salicylate (SS, 200 mg/kg) conditions. Gap thresholds could not be measured at 20 dB SPL during salicylate treatment. **(B)** Mean (SEM) gap detection thresholds for the current saline and salicylate (SS, 200 mg/kg) data re-plotted in dB SL (sensation level). Mean (SEM) gap detection thresholds for white noise are also plotted for the Mongolian gerbil across five sensation levels (35).

Figure 1A shows the mean BBN gap thresholds at 30, 40, and 60 dB SPL for all six rats for baseline, saline, and salicylate conditions. In addition, the gap thresholds for baseline and saline are shown for 20 dB SPL; however, we were unable to collect reliable data for salicylate at 20 dB SPL. For baseline and saline conditions, mean BBN gap detection thresholds increased slightly from ~2 to 3 ms as the sound level decreased from 60 to 30 dB SPL, but at 20 dB SPL, gap thresholds increased to 4–6 ms. A two-way repeated measures analysis of variance (ANOVA) of the baseline and saline gap threshold obtained at 20–60 dB SPL showed a significant effect of intensity (*F* = 15.77, 3 df; *p* < 0.001) and a significant interaction between intensity and baseline and saline conditions (*F* = 11.79, 3 df, *p* < 0.001). A Holm–Sidak multiple comparison of the baseline condition revealed a significant difference in gap thresholds between 20 and 60 dB and 20 and 40 dB (*p* < 0.01); a similar analysis of the saline condition revealed a significant difference between 20 and 30 dB, 20 and 40 dB, and 20 and 60 dB (*p* < 0.05).

Mean gap thresholds during salicylate treatment were similar to baseline and saline at 60 dB SPL (Figure 1A; Table 1). At 40 dB SPL, mean gap thresholds increased from 2 ms pre-treatment to about 3 ms during salicylate; however, at 30 dB SPL, gap detection thresholds increased from ~3 ms pre-treatment to roughly 10 ms during salicylate, but did not exceed 18 ms in any of the salicylate-treated rats (Table 1; range: 3.9–17.7 ms).

A two-way repeated measures ANOVA with three sound levels (30, 40, and 60 dB SPL) and three conditions (baseline, saline, and salicylate) as factors revealed a significant effect of condition (*F* = 14.77, 2 df, *p* < 0.001), intensity (*F* = 16.50, 2 df, *p* < 0.001), and a significant interaction between condition and sound level (*F* = 9.53, 4 df, *p* < 0.001). A Holm–Sidak *post hoc* analysis revealed significant differences between the salicylate and saline conditions (*p* < 0.05) at 30 dB SPL and between salicylate and baseline conditions (*p* < 0.05) at 30 dB SPL, but not at 40 and 60 dB SPL (*p* > 0.05). Within the saline treatment group, the Holm–Sidak *post hoc* analysis revealed a significant difference between 30 and 60 dB SPL (*p* < 0.05) and 30 and 40 dB SPL (*p* < 0.05). No significant differences were found between the baseline and saline conditions for any of the sound levels (*p* > 0.05).

Mean BBN gap detection thresholds for the saline and salicylate conditions were subsequently re-plotted by sensation level (dB SL) in Figure 1B to more clearly illustrate the effects of salicylate-induced hearing loss on gap detection performance. The mean SLs were calculated by subtracting the mean thresholds from the noise-burst detection experiment (dB SPL) from the sound presentation level of the stimulus. In addition, gap detection thresholds were also plotted for normal-hearing Mongolian gerbils across five sensation levels (35). The reason for including the gerbil data was to demonstrate that gap detection deficits are apparent with decreasing sound level in normal-hearing animals with similar hearing sensitivity and temporal processing abilities as rats. Therefore, when corrected for hearing loss and re-plotted by sensation level, the gap detection deficits seen following an injection of salicylate appear to be the result of hearing loss alone without the additional explanation of tinnitus “filling in” the silent gap.

#### Gap detection in narrowband noise

**Figure 2:**
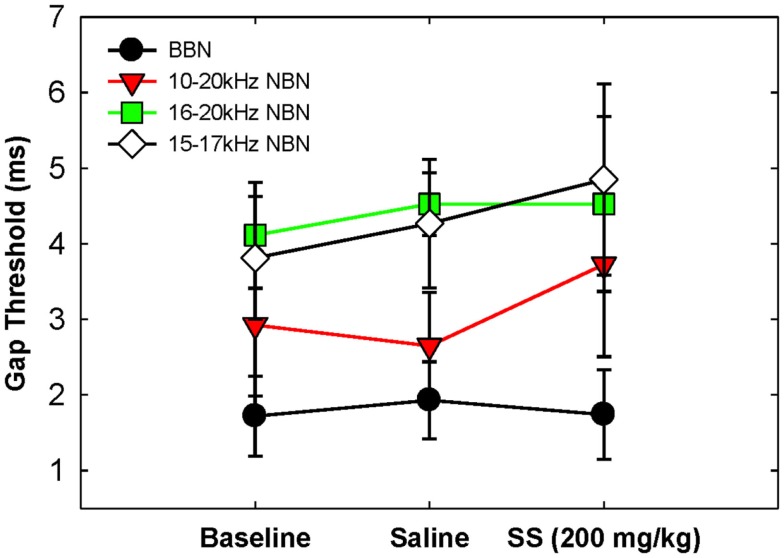
**Mean (SEM) gap thresholds for BBN and three NBNs obtained for baseline, saline, and salicylate (SS, 200 mg/kg) conditions**.

**Figure 3:**
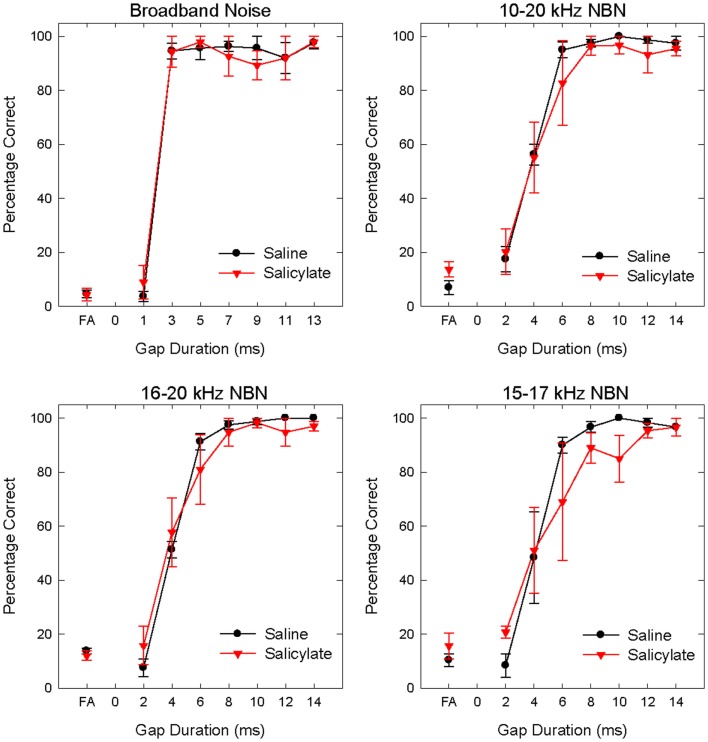
**Mean (SEM) psychometric functions in the BBN (60 dB SPL) and NBN (60 dB SPL) gap detection experiments for saline and salicylate (SS, 200 mg/kg) conditions**. False alarm (FA) rates are also plotted.

Gap detection thresholds were also obtained at 60 dB SPL for three NBNs (10–20, 16–20, and 15–17 kHz) located near the most sensitive region of the rat’s audiogram and in the vicinity of the presumed tinnitus pitch (7, 19, 39). The purpose of this experiment was to determine if salicylate-induced tinnitus could “fill-in” the silent gap in NBN. Table 2 shows the gap detection thresholds in BBN and the three NBNs at 60 dB SPL for each rat across the baseline, saline, and salicylate conditions. Mean thresholds for the four rats are plotted in Figure 2 (two of the six rats previously tested in the BBN condition died before their gap detection thresholds in NBN could be obtained), and mean psychometric functions are plotted in Figure 3. Generally, gap detection thresholds increased as the bandwidth of the noise became narrower; however, gap detection thresholds for each NBN did not change significantly after the rats were injected with salicylate.

**Table 2:** **Individual and mean gap detection thresholds (ms) for broadband noise (BBN) and narrowband noise (NBN) at 60 dB SPL**.

Subject	Condition	BBN (60 dB SPL)	10–20 kHz NBN (60 dB SPL)	16–20 kHz NBN (60 dB SPL)	15–17 kHz NBN (60 dB SPL)
Green 1	Baseline	2.1 ms	2.05 ms	4.4 ms	3.4 ms
	Saline	1.8 ms	2.5 ms	4.3 ms	NA
	Salicylate	2.0 ms	2.9 ms	3.7 ms	3.4 ms
Green 3	Baseline	1.8 ms	2.5 ms	3.95 ms	3.5 ms
	Saline	1.9 ms	2.7 ms	4.5 ms	3.9 ms
	Salicylate	NA	2.6 ms	3.7 ms	3.9 ms
Purple 2	Baseline	1.5 ms	3.05 ms	3.5 ms	3.55 ms
	Saline	1.7 ms	2.1 ms	4.6 ms	3.8 ms
	Salicylate	1.6 ms	5.9 ms	6.5 ms	7.0 ms
Purple 3	Baseline	1.75 ms	4.1 ms	4.6 ms	4.8 ms
	Saline	1.93 ms	3.3 ms	4.7 ms	5.1 ms
	Salicylate	1.74 ms	3.5 ms	4.2 ms	5.1 ms
Mean	Baseline	1.72 ms	2.93 ms	4.11 ms	3.81 ms
	Saline	1.93 ms	2.65 ms	4.53 ms	4.27 ms
	Salicylate	1.74 ms	3.73 ms	4.53 ms	4.85 ms

*NA, data not available*.

In a two-way repeated measures ANOVA with three conditions (baseline, saline, and salicylate) and four noise bandwidths (BBN, 10–20, 16–20, and 15–17 kHz) as factors, the gap threshold differences among the three conditions were not great enough to exclude the possibility that the differences were due to chance [*F*(2,45) = 0.42, *p* > 0.05], and there was no significant interaction between condition and bandwidth [*F*(6,45) = 2.16, *p* > 0.05].

### Noise thresholds

**Figure 4:**
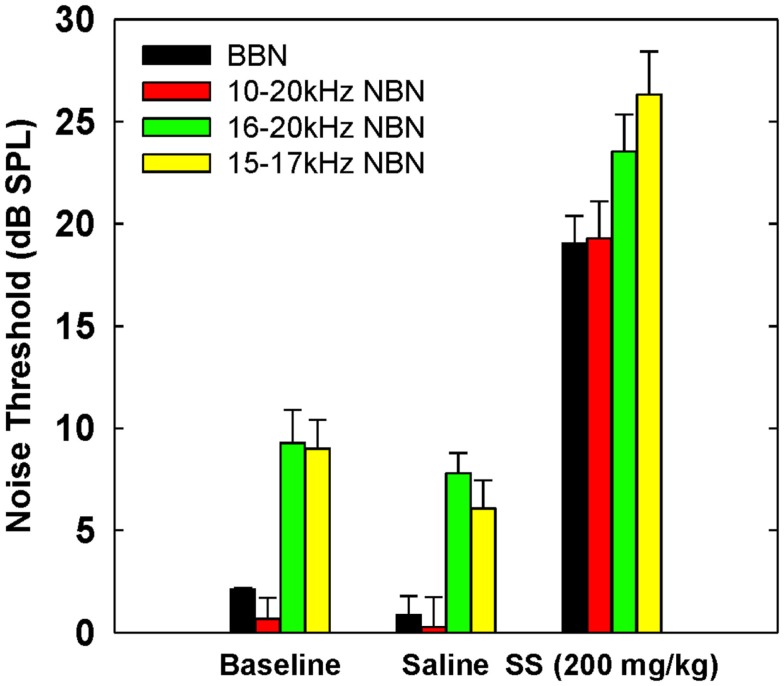
**Mean (SEM) thresholds for BBN and three NBN bandwidths (see legend) during baseline, saline and salicylate (SS, 200 mg/kg) conditions for three rats**. BBN data from one rat, Purple 2, were not shown in this graph because her thresholds could not be collected for the NBN conditions.

Table 3 shows broadband and NBN thresholds in quiet for each of the three rats that were tested in the baseline, saline, and salicylate conditions (Note: two of the original six rats died before noise thresholds could be obtained and one rat had a broken tooth during threshold testing so complete thresholds are shown for three rats). Since one rat, Purple 2, could not perform in the NBN threshold conditions due to a tooth problem, mean performance for the other three rats is plotted in Figure 4 and statistics were only carried out using the three rats that could complete all threshold conditions. However, BBN thresholds for rat Purple 2 were included in Table 3 for comparison.

**Table 3:** **Individual broadband noise (BBN) and narrowband noise (NBN) thresholds**.

Subject	Condition	BBN	10–20 kHz NBN	16–20 kHz NBN	15–17 kHz NBN
Green 1	Baseline	2.6 dB SPL	1.6 dB SPL	6.8 dB SPL	11.3 dB SPL
	Saline	1.2 dB SPL	2.7 dB SPL	6.7 dB SPL	7.7 dB SPL
	Salicylate	17.7 dB SPL	16.1 dB SPL	26.3 dB SPL	30.5 dB SPL
Green 3	Baseline	2.0 dB SPL	−0.5 dB SPL	12.0 dB SPL	8.1 dB SPL
	Saline	1.5 dB SPL	−0.7 dB SPL	8.1 dB SPL	6.5 dB SPL
	Salicylate	21.1 dB SPL	22.6 dB SPL	24.5 dB SPL	26.8 dB SPL
Purple 2	Baseline	4.8 dB SPL	NA	NA	NA
	Saline	7.1 dB SPL	NA	NA	NA
	Salicylate	24.7 dB SPL	NA	NA	NA
Purple 3	Baseline	1.8 dB SPL	1.0 dB SPL	9.2 dB SPL	7.7 dB SPL
	Saline	−0.1 dB SPL	−1.2 dB SPL	8.6 dB SPL	4.0 dB SPL
	Salicylate	18.3 dB SPL	19.2 dB SPL	19.8 dB SPL	21.7 dB SPL
Mean	Baseline	2.80 dB SPL	0.67 dB SPL	9.28 dB SPL	9.00 dB SPL
	Saline	2.43 dB SPL	0.27 dB SPL	7.80 dB SPL	6.07 dB SPL
	Salicylate	20.45 dB SPL	19.30 dB SPL	23.53 dB SPL	26.33 dB SPL

*According to this table, a 20 dB SPL BBN following an injection of salicylate would only be 2.3 and 1.7 dB above threshold for green 1 and purple 3, respectively; the BBN would be below threshold for Purple 2 and Green 3*.

*NA, data not available*.

Mean thresholds were the lowest for BBN and the 10–20 kHz NBN and 7–9 dB higher for the 10–20 and 15–17 kHz NBN. Noise thresholds for BBN, 10–20 kHz NBN, 16–20 kHz NBN, and 15–17 NBN during the salicylate condition increased ~18, 19, 16, and 20 dB, respectively, compared to the saline control condition. A two-way repeated measures ANOVA with three conditions (baseline, saline, and salicylate) and four noise bandwidths (BBN, 10–20, 16–20, and 15–17 kHz) as factors revealed significant main effects of condition [*F*(2,35) = 322.8, *p* < 0.001] and noise bandwidth [*F*(3,35) = 26.2, *p* < 0.001], but there was no significant interaction between condition and bandwidth [*F*(6,35) = 0.80, *p* > 0.05]. Holm–Sidak *post hoc* tests found significant differences between the salicylate and saline conditions (*p* < 0.05), and the salicylate and baseline conditions (*p* < 0.05) for all four noises, with the salicylate condition having significantly higher thresholds than the baseline and saline conditions. No significant differences were found between the baseline and saline conditions for any of the noises (*p* > 0.05). The *post hoc* analyses also revealed significant differences between BBN and the 16–20 and 15–17 kHz NBNs (*p* < 0.05), but not the 10–20 kHz NBN (*p* > 0.05). Overall, the 16–20 and 15–17 kHz NBNs consistently produced higher thresholds than the BBN and 10–20 kHz NBN.

## Discussion

### Normal gap detection thresholds in rats

Most gap detection studies employ BBN stimuli to minimize problems associated with spectral splatter and off-frequency listening (40, 41). Our baseline BBN gap detection thresholds in Sprague-Dawley rats were in the 2–3 ms range from 30 to 60 dB SPL (~28–58 dB SL). However, at 20 dB SPL (~18 dB SL) mean gap thresholds increased to 4.3 ms. Our BBN gap thresholds are in good agreement to those previously reported in rats (42, 43), mice (37, 44), chinchillas (45), gerbils (35), and humans (13, 40, 46), and show a clear increase as intensity falls below 30 dB SPL.

Our NBN gap thresholds measured at 60 dB SPL were larger than those obtained in BBN (Figure 2; Table 2). The gap thresholds for the two narrowest NBNs (16–20 and 15–17 kHz) were ~1 ms longer than the 10–20 kHz NBN gap threshold, which in turn was roughly 1 ms longer than the BBN. These results indicate that gap thresholds improve (decrease) as stimulus bandwidth increases. Few animal studies have examined the role of stimulus bandwidth on gap detection performance (37, 47) and, to date, no studies have examined the role of frequency and bandwidth on gap detection in rats. However, our results are consistent with human studies that show an improvement in gap thresholds with increasing stimulus bandwidth (48–53).

### Effects of salicylate on gap detection thresholds

The main goal of the current project was to determine if a dose of salicylate sufficient to induce hearing loss and tinnitus would significantly impair gap detection performance in rats. However, since previous research in normal-hearing humans (40) and animals (35, 37, 45) has found that gap detection thresholds increase significantly with decreasing stimulus intensity, hearing loss is a primary confounding variable when assessing gap detection thresholds in hearing-impaired listeners. In other words, for all species tested, gap detection thresholds increase with decreasing sound level, regardless of tinnitus or any other temporal processing deficit (35, 40). As a result, the hearing loss induced by salicylate needs to be considered when evaluating our gap detection results.

Because the 200 mg/kg dose of salicylate increased the BBN threshold from 2 to 19 dB SPL (Table 3), it was not technically feasible to measure gap thresholds with the 20 dB SPL BBN due to the fact that the signal was essentially undetectable at this intensity. However, gap thresholds increased to 10 ms at 30 dB SPL (Figure 1); though this increase is most likely due to the fact that the BBN was now only 10 dB above threshold versus ~28 dB above threshold before salicylate treatment (Table 3). These results are in line with previous research in animals (35, 37, 45) and humans (13, 40) showing increased gap detection thresholds with decreasing stimulus intensity (Figure 1B).

Gap detection thresholds at 40 and 60 dB SPL were largely unaffected by salicylate treatment. Likewise, NBN gap detection thresholds measured at 60 dB SPL were largely unaffected by salicylate treatment; mean gap thresholds increased by <1.08 ms post-salicylate when compared to saline values (Figure 2), and salicylate thresholds only appeared to increase in one rat, Purple 2 (Table 2). However, since NBN threshold measurements could not be taken from this rat due to illness, it is possible that this rat had more hearing loss as a result of salicylate treatment than the other rats. This rat’s (Purple 2) BBN threshold following salicylate administration was the highest of all four rats tested and was at least 3.6 dB SPL higher than the next highest threshold (Table 3). Taken together, our results mirror gap detection data in humans indicating that salicylate had little or no effect on the detection of silent gaps embedded in BBN or NBN as long as the signals remained audible and well above threshold (13).

Even under the most difficult listening conditions post-salicylate (30 dB SPL BBN), gap thresholds never exceeded 18 ms in any of the animals tested (Table 1). The changes in gap detection we observed in rats treated with 200 mg/kg salicylate are similar to those observed in humans treated with a high doses of aspirin, whose active ingredient is salicylate (13). The dose of aspirin employed in the McFadden et al. (13) study-induced hearing losses ranging from 4 to 18 dB in humans. Gap detection thresholds, assessed with low- and high-frequency octave-band noise, were unaffected by the aspirin-induced hearing loss at moderate and high intensities, but increased by ~15 ms at low intensities. Therefore, as in the McFadden et al. (13) study, the increase in gap thresholds at low SPLs in our experiment is most likely the result of the salicylate-induced hearing loss rather than an impairment in temporal resolution caused by tinnitus “filling in” the conscious perception of the silent gap.

### Tinnitus gap-filling hypothesis

In addition to providing important animal psychoacoustic data, our results also have implications for tinnitus research. One of the most widely used animal behavioral models of tinnitus, Gap-PPI, operates on the hypothesis that tinnitus interferes with processing silent gaps in noise (19, 21–23, 29, 31). Most Gap-PPI paradigms employ a 50 ms silent gap embedded in an ongoing 60 dB SPL background noise to suppress the acoustic startle reflex response. Gap stimuli embedded in BBN or NBN strongly inhibit the startle response; but, if an animal is exposed to intense noise or given a high dose of salicylate, the silent gap no longer suppresses the startle response. This lack of prepulse inhibition has been hypothesized to occur because the phantom sound of tinnitus “fills in” the 50 ms silent intervals in the noise thereby preventing the silent gap from suppressing the subsequent acoustic startle reflex (19–21, 24, 25, 31).

However, this “tinnitus gap-filling” hypothesis has been evaluated in only three human studies to date. Two of these human studies used psychoacoustic procedures to determine whether tinnitus interferes with the *conscious* perception of silent gaps in noise. Campolo et al. (30) found that when tinnitus patients with pre-existing hearing loss were asked if they could detect 50 ms silent gaps embedded in 15 dB SL NBN that was centered above, below, or at their tinnitus frequency, tinnitus patients had no difficulty detecting the 50 ms gaps and performed as well as normal-hearing subjects (30). More recently, Boyen et al. (54) found that human tinnitus patients had similar gap detection thresholds compared to a matched non-tinnitus control group even when the test frequency matched the patient’s tinnitus frequency (54).

The third recent human study used an eye-blink Gap-PPI paradigm to test gap detection in tinnitus patients (55). Fournier and Hébert (55) found that patients with high-frequency hearing loss and high-pitched tinnitus had less Gap-PPI than normal controls at high frequencies, but they also had less Gap-PPI at low frequencies where there was no tinnitus. If the high-pitched tinnitus was filling in the silent gap, the authors reasoned that the tinnitus patients should have impaired Gap-PPI only at the high frequencies and not the low frequencies (55). Furthermore, since the tinnitus group in the Fournier and Hébert (55) study had a high-frequency hearing loss while the control group did not, it is not surprising that their tinnitus subjects would show greater gap detection deficits than the control subjects given that previous research has shown that high-frequency information is important for sensitive gap detection performance. When high frequencies are unavailable to a subject, either through a hearing loss or through filtering of the test stimuli, gap detection thresholds increase (15, 37, 40, 42, 48).

Our current psychophysical results provide the first test of the “tinnitus gap-filling” hypothesis in an animal. The results of our gap detection experiment mirror the results found in the human psychophysical gap detection tests (13, 30, 54), and suggest that tinnitus does not “fill-in” the silent gap in tasks requiring conscious perception.

### The confounding effects of salicylate and noise exposure

One of the difficulties in developing an animal behavioral model of tinnitus is controlling for the confounding effects of the tinnitus inducer, i.e., salicylate or noise exposure (18, 56). In addition to tinnitus, salicylate is also known to induce hearing loss in humans and animals (1, 13), temporal resolution and integration deficits in humans (13), and hyperacusis in animals (22, 57). Therefore, any animal behavioral paradigm must account for these confounding variables when assessing animals for tinnitus.

Researchers using Gap-PPI, including our lab, have attempted to account for salicylate-induced hearing loss and gap detection deficits by using background noises of at least 60 dB SPL and 50 ms silent gaps (19–21, 24). Using these, and similar, stimulus parameters, researchers have consistently found Gap-PPI deficits, i.e., reduced inhibition, in animals following salicylate exposure and have attributed these deficits to the presence of tinnitus (19, 20). However, the results of the current experiment, along with recent human psychoacoustic and startle reflex data, call into question what the Gap-PPI paradigm is modeling (32). If hearing loss and gap detection deficits are not producing these changes in Gap-PPI because the stimuli are presented at high sound levels (~60 dB SPL), with long-duration gaps (~50 ms), and if tinnitus is not filling in the silent gaps, as suggested by the current results and recent human data (30, 54, 55), then it is not yet clear what is producing Gap-PPI deficits following salicylate and noise exposure.

One explanation for the changes in Gap-PPI following salicylate or noise exposure could be related to hyperacusis. Briefly, hyperacusis is defined as abnormal sound level tolerance where moderate-level sounds are perceived as uncomfortably loud (58, 59). Both salicylate and noise exposure have recently been implicated in the manifestation of hyperacusis in animals. Salicylate has been shown to amplify acoustic startle responses (ASRs) (8, 20) and alter loudness growth functions to moderate- and high-level sounds (57) in rats. In addition, noise exposures that result in either permanent threshold shifts (22) or only temporary threshold shifts (60) have been shown to amplify ASRs in animals, which may be indicative of hyperacusis (18). Given these consistent changes to ASRs following salicylate and noise exposure, this could be a confounding variable when measuring Gap-PPI because PPI is not independent from baseline startle reactivity (61, 62).

Another explanation for changes in Gap-PPI following salicylate are alterations in temporal processing. Sun et al. (63) found that salicylate facilitates the perception of the onset of the gap, but reduces the perception of the offset of the gap. In other words, salicylate increases Gap-PPI by facilitating the response to the onset of the gaps, while decreasing Gap-PPI when the offset of the gap is the available cue (63). Since salicylate *facilitated* the ASR, (63) determined that these changes were not due to hearing loss. However, since temporal integration is altered in humans given large doses of aspirin (13), and since salicylate enhances the sound-evoked onset responses of cortical neurons (9), it is possible that changes in temporal integration following salicylate could explain the Sun et al. (63) results, regardless of tinnitus.

Although the Gap-PPI paradigm has many advantages over other animal models of tinnitus, it is not yet clear that tinnitus alone can account for changes in Gap-PPI when both salicylate and noise exposure appear to induce hearing loss, temporal processing deficits, and hyperacusis as well (18, 32).

## Concluding Remarks

In summary, the gap detection thresholds for rats in the current study were unchanged with salicylate for BBN and NBN presented at high-sound levels, suggesting that salicylate-induced tinnitus was not “filling in” the conscious perception of the silent gaps. Furthermore, given the threshold shifts found in the noise detection experiments, the effects of salicylate on gap detection at low-sound levels are most parsimoniously explained by hearing loss alone and not caused by tinnitus. However, the possibility remains that tinnitus interferes with peripheral, pre-attentive filtering of sensory stimuli involved in sensorimotor gating. As such, the conscious perception of silent gaps in background noise may involve different neural processes than those underlying prepulse inhibition of the acoustic startle reflex; however, hearing loss, temporal processing deficits, and hyperacusis remain confounding variables when using traditional gap detection paradigms to measure tinnitus in animals.

## Author Contributions

KR, DS, and RS conceptualized and designed the experiment. KR, MU, and RB collected and analyzed the data. KR and RS interpreted the data and wrote the manuscript. KR, DS, MU, RB, and RS revised the manuscript and approved the final version.

## Conflict of Interest Statement

The authors declare that the research was conducted in the absence of any commercial or financial relationships that could be construed as a potential conflict of interest.

## References

[1] Brennan JF Brown CA Jastreboff PJ. Salicylate-induced changes in auditory thresholds of adolescent and adult rats. Dev Psychobiol (1996) 29:69–86. 10.1002/(SICI)1098-2302(199601)29:1<69::AID-DEV4>3.3.CO;2-R 8719183

[2] Cazals Y. Auditory sensori-neural alterations induced by salicylate. Prog Neurobiol (2000) 62:583–631. 10.1016/S0301-0082(00)00027-7 10880852

[3] Chen GD Stolzberg D Lobarinas E Sun W Ding D Salvi R. Salicylate-induced cochlear impairments, cortical hyperactivity and re-tuning, and tinnitus. Hear Res (2013) 295:100–13. 10.1016/j.heares.2012.11.016 23201030 PMC4191647

[4] Stolzberg D Hayes SH Kashanian N Radziwon K Salvi RJ Allman BL. A novel behavioral assay for the assessment of acute tinnitus in rats optimized for simultaneous recording of oscillatory neural activity. J Neurosci Methods (2013) 219:224–32. 10.1016/j.jneumeth.2013.07.021 23933328 PMC3796950

[5] Sheppard A Hayes SH Chen G-D Ralli M Salvi R. Review of salicylate-induced hearing loss, neurotoxicity, tinnitus and neuropathophysiology. Acta Otorhinolaryngol Ital (2014) 34:79–93. 24843217 PMC4025186

[6] Chen G-D Kermany MH D’Elia A Ralli M Tanaka C Bielefeld EC Too much of a good thing: long-term treatment with salicylate strengthens outer hair cell function but impairs auditory neural activity. Hear Res (2010) 265:63–9. 10.1016/j.heares.2010.02.010 20214971 PMC4191643

[7] Stolzberg D Chen GD Allman BL Salvi RJ. Salicylate-induced peripheral auditory changes and tonotopic reorganization of auditory cortex. Neuroscience (2011) 180:157–64. 10.1016/j.neuroscience.2011.02.005 21310217 PMC3070811

[8] Sun W Lu J Stolzberg D Gray L Deng A Lobarinas E Salicylate increases the gain of the central auditory system. Neuroscience (2009) 159:325–34. 10.1016/j.neuroscience.2008.12.024 19154777 PMC2759817

[9] Stolzberg D Chrostowski M Salvi RJ Allman BL. Intracortical circuits amplify sound-evoked activity in primary auditory cortex following systemic injection of salicylate in the rat. J Neurophysiol (2012) 108:200–14. 10.1152/jn.00946.2011 22496535 PMC3434608

[10] Ochi K Eggermont JJ. Effects of salicylate on neural activity in cat primary auditory cortex. Hear Res (1996) 95:63–76. 10.1016/0378-5955(96)00019-6 8793509

[11] Johnson NJ Elberling C. Evoked acoustic emissions from the human ear: I. Equipment and response parameters. Scand Audiol (1982) 11:3–12. 10.3109/01050398209076194 7178800

[12] McFadden D Plattsmier HS. Aspirin can potentiate the temporary hearing loss induced by intense sounds. Hear Res (1983) 9:295–316. 10.1016/0378-5955(83)90033-3 6841285

[13] McFadden D Plattsmier HS Pasanen EG. Aspirin-induced hearing loss as a model of sensorineural hearing loss. Hear Res (1984) 16:251–60. 10.1016/0378-5955(84)90114-X 6401084

[14] McFadden D Wightman FL Audition: some relations between normal and pathological hearing. Annu Rev Psychol (1983) 34:95–128 10.1146/annurev.ps.34.020183.000523 6338813

[15] Salvi RJ Arehole S. Gap detection in chinchillas with temporary high-frequency hearing loss. J Acoust Soc Am (1985) 77:1173–7. 10.1121/1.392181 3980869

[16] Jastreboff PJ Brennan JF Coleman JK Sasaki CT. Phantom auditory sensation in rats: an animal model for tinnitus. Behav Neurosci (1988) 102:811–22. 10.1037/0735-7044.102.6.811 3214530

[17] von der Behrens W Animal models of subjective tinnitus. Neural Plast (2014) 2014:741452 10.1155/2014/741452 24829805 PMC4009209

[18] Hayes SH Radziwon KE Stolzberg DJ Salvi RJ. Behavioral models of tinnitus and hyperacusis in animals. Front Neurol (2014) 5:179. 10.3389/fneur.2014.00179 25278931 PMC4166233

[19] Yang G Lobarinas E Zhang L Turner J Stolzberg D Salvi R Salicylate induced tinnitus: behavioral measures and neural activity in auditory cortex of awake rats. Hear Res (2007) 226:244–53. 10.1016/j.heares.2006.06.013 16904853

[20] Turner JG Parrish J. Gap detection methods for assessing salicylate-induced tinnitus and hyperacusis in rats. Am J Audiol (2008) 17:S185–92. 10.1044/1059-0889(2008/08-0006) 18978200

[21] Longenecker RJ Galazyuk AV. Development of tinnitus in CBA/CaJ mice following sound exposure. J Assoc Res Otolaryngol (2011) 12:647–58. 10.1007/s10162-011-0276-1 21667173 PMC3173549

[22] Chen G Lee C Sandridge SA Butler HM Manzoor NF Kaltenbach JA. Behavioral evidence for possible simultaneous induction of hyperacusis and tinnitus following intense sound exposure. J Assoc Res Otolaryngol (2013) 14:413–24. 10.1007/s10162-013-0375-2 23440516 PMC3642276

[23] Pace E Zhang J. Noise-induced tinnitus using individualized gap detection analysis and its relationship with hyperacusis, anxiety, and spatial cognition. PLoS One (2013) 8:e75011. 10.1371/journal.pone.0075011 24069375 PMC3771890

[24] Berger JI Coomber B Wells TT Wallace MN Palmer AR. Changes in the response properties of inferior colliculus neurons relating to tinnitus. Front Neurol (2014) 5:203. 10.3389/fneur.2014.00203 25346722 PMC4191193

[25] Coomber B Berger JI Kowalkowski VL Shackleton TM Palmer AR Wallace MN. Neural changes accompanying tinnitus following unilateral acoustic trauma in the guinea pig. Eur J Neurosci (2014) 40:2427–41. 10.1111/ejn.12580 24702651 PMC4215599

[26] Ison JR. Temporal acuity in auditory function in the rat: reflex inhibition by brief gaps in noise. J Comp Physiol Psychol (1982) 96:945–54. 10.1037/0735-7036.96.6.945 7153390

[27] Ison JR Taylor MK Bowen GP Schwarzkopf SB. Facilitation and inhibition of the acoustic startle reflex in the rat after a momentary increase in background noise level. Behav Neurosci (1997) 111:1335–52. 10.1037/0735-7044.111.6.1335 9438802

[28] Cooper JCJ. Health and nutrition examination survey of 1971-75: Part II. Tinnitus, subjective hearing loss, and well-being. J Am Acad Audiol (1994) 5:37–43. 8155893

[29] Turner JG Brozoski TJ Bauer CA Parrish JL Myers K Hughes LF Gap detection deficits in rats with tinnitus: a potential novel screening tool. Behav Neurosci (2006) 120:188–95. 10.1037/0735-7044.120.1.188 16492129

[30] Campolo J Lobarinas E Salvi R. Does tinnitus “fill in” the silent gaps? Noise Health (2013) 15:398–405. 10.4103/1463-1741.121232 24231418 PMC3875329

[31] Dehmel S Eisinger D Shore SE. Gap prepulse inhibition and auditory brainstem-evoked potentials as objective measures for tinnitus in guinea pigs. Front Syst Neurosci (2012) 6:42. 10.3389/fnsys.2012.00042 22666193 PMC3364697

[32] Eggermont JJ. Hearing loss, hyperacusis, or tinnitus: what is modeled in animal research? Hear Res (2013) 295:140–9. 10.1016/j.heares.2012.01.005 22330978

[33] Lobarinas E Sun W Cushing R Salvi R. A novel behavioral paradigm for assessing tinnitus using schedule-induced polydipsia avoidance conditioning (SIP-AC). Hear Res (2004) 190:109–14. 10.1016/S0378-5955(04)00019-X 15051133

[34] Steckler T. Using signal detection methods for analysis of operant performance in mice. Behav Brain Res (2001) 125:237–48. 10.1016/S0166-4328(01)00305-9 11682115

[35] Wagner E Klump GM Hamann I. Gap detection in Mongolian gerbils (*Meriones unguiculatus*). Hear Res (2003) 176:11–6. 10.1016/S0378-5955(02)00643-3 12583877

[36] Klink KB Bendig G Klump GM Operant methods for mouse psychoacoustics. Behav Res Methods (2006) 38:1–7 10.3758/BF03192744 16817508

[37] Radziwon KE June KM Stolzberg DJ Xu-Friedman MA Salvi RJ Dent ML. Behaviorally measured audiograms and gap detection thresholds in CBA/CaJ mice. J Comp Physiol A Neuroethol Sens Neural Behav Physiol (2009) 195:961–9. 10.1007/s00359-009-0472-1 19756650 PMC2813807

[38] Klump GM Maier EH. Gap detection in the starling (*Sturnus vulgaris*). J Comp Physiol A (1989) 164:531–8. 10.1016/j.visres.2010.09.020 20875443

[39] Brennan JF Jastreboff PJ. Generalization of conditioned suppression during salicylate-induced phantom auditory perception in rats. Acta Neurobiol Exp (1991) 51:15–27. 1759596

[40] Fitzgibbons PJ. Temporal gap resolution in narrow-band noises with center frequencies from 6000-14000 Hz. J Acoust Soc Am (1984) 75:566–9. 10.1121/1.390529 6699295

[41] Shailer MJ Moore BCJ. Detection of temporal gaps in bandlimited noise: effects of variations in bandwidth and signal-to-masker ratio. J Acoust Soc Am (1985) 77:635–9. 10.1121/1.391881 3973235

[42] Syka J Rybalko N Mazelová J Druga R. Gap detection threshold in the rat before and after auditory cortex ablation. Hear Res (2002) 172:151–9. 10.1016/S0378-5955(02)00578-6 12361878

[43] Rybalko N Syka J. Effect of noise exposure on gap detection in rats. Hear Res (2005) 200:63–72. 10.1016/j.heares.2004.08.014 15668039

[44] Walton JP Frisina RD Ison JR O’Neill WE. Neural correlates of behavioral gap detection in the inferior colliculus of the young CBA mouse. J Comp Physiol A (1997) 181:161–76. 10.1007/s003590050103 9251257

[45] Giraudi D Salvi R Henderson D Hamernik R. Gap detection by the chinchilla. J Acoust Soc Am (1980) 68:802–6. 10.1121/1.384818 7419814

[46] Plomp R Rate of decay of auditory sensation. J Acoust Soc Am (1964) 36:277–82 10.1121/1.1918946

[47] Ison JR Allen PD Rivoli PJ Moore JT. The behavioral response of mice to gaps in noise depends on its spectral components and its bandwidth. J Acoust Soc Am (2005) 117:3944–51. 10.1121/1.1904387 16018496

[48] Buus S Florentine M Gap detection in normal and impaired listeners: the effect of level and frequency. In: Michelsen A, editor. Time Resolution in Auditory Systems. New York, NY: Springer-Verlag (1985). p. 159–79.

[49] Formby C Muir K. Modulation and gap detection for broadband and filtered noise signals. J Acoust Soc Am (1988) 84:545–50. 10.1121/1.396831 3170945

[50] Grose JH Eddins DA Hall JWI. Gap detection as a function of stimulus bandwidth with fixed high-frequency cutoff in normal-hearing and hearing-impaired listeners. J Acoust Soc Am (1989) 86:1747–55. 10.1121/1.398606 2808923

[51] Grose JH. Gap detection in multiple narrow bands of noise as a function of spectral configuration. J Acoust Soc Am (1991) 90:3061–8. 10.1121/1.401780 1787244

[52] Eddins DA Hall JWI Grose JH. The detection of temporal gaps as a function of frequency region and absolute noise bandwidth. J Acoust Soc Am (1992) 91:1069–77. 10.1121/1.402633 1556308

[53] Snell KB Ison JR Frisina DR. The effects of signal frequency and absolute bandwidth on gap detection in noise. J Acoust Soc Am (1994) 96:1458–64. 10.1121/1.410288 7963009

[54] Boyen K Baskent D van Dijk P Gap detection thresholds in tinnitus subjects: does tinnitus fill in the silent gaps? 8th International TRI Tinnitus Conference Auckland (2014).

[55] Fournier P Hébert S. Gap detection deficits in humans with tinnitus as assessed with the acoustic startle paradigm: does tinnitus fill in the gap? Hear Res (2013) 295:16–23. 10.1016/j.heares.2012.05.011 22688322

[56] Heffner HE Heffner RS Behavioral tests for tinnitus in animals. In: Eggermont JJ Zeng F-G Popper AN Fay RR, editors. Tinnitus. New York, NY: Springer Science+Business Media (2012). p. 21–58.

[57] Chen G-D Radziwon KE Kashanian N Manohar S Salvi R. Salicylate-induced auditory perceptual disorders and plastic changes in nonclassical auditory centers in rats. Neural Plast (2014) 2014:658741. 10.1155/2014/658741 24891959 PMC4033555

[58] Blaesing L Kroener-Herwig B. Self-reported and behavioral sound level avoidance in tinnitus and hyperacusis subjects, and association with anxiety ratings. Int J Audiol (2012) 51:611–7. 10.3109/14992027.2012.664290 22443320

[59] Tyler RS Pienkowski M Roncancio ER Jun HJ Brozoski T Dauman N A review of hyperacusis and future directions: part I. Definitions and manifestations. Am J Audiol (2014) 23(4):402–19. 10.1044/2014_AJA-14-0010 25104073

[60] Hickox AE Liberman MC. Is noise-induced cochlear neuropathy key to the generation of hyperacusis or tinnitus? J Neurophysiol (2014) 111:552–64. 10.1152/jn.00184.2013 24198321 PMC3921399

[61] Csomor PA Yee BK Vollenweider FX Feldon J Nicolet T Quednow BB. On the influence of baseline startle reactivity on the indexation of prepulse inhibition. Behav Neurosci (2008) 122:885–900. 10.1037/0735-7044.122.4.885 18729642

[62] Lobarinas E Hayes SH Allman BL. The gap-startle paradigm for tinnitus screening in animal models: limitations and optimization. Hear Res (2013) 295:150–60. 10.1016/j.heares.2012.06.001 22728305 PMC3505812

[63] Sun W Doolittle L Flowers E Zhang C Wang Q. High doses of salicylate causes prepulse facilitation of onset-gap induced acoustic startle response. Behav Brain Res (2014) 258:187–92. 10.1016/j.bbr.2013.10.024 24149068

